# Association of Hantavirus Infections and Leptospirosis With the Occurrence of Chronic Kidney Disease of Uncertain Etiology in the North Central Province of Sri Lanka: A Prospective Study With Patients and Healthy Persons

**DOI:** 10.3389/fcimb.2020.556737

**Published:** 2020-10-08

**Authors:** N. P. Sunil-Chandra, J. A. A. S. Jayaweera, W. Kumbukgolla, M. V. M. L. Jayasundara

**Affiliations:** ^1^Department of Medical Microbiology, Faculty of Medicine, University of Kelaniya, Kelaniya, Sri Lanka; ^2^Faculty of Medicine & Allied Sciences, Rajarata University of Sri Lanka, Anuradhapura, Sri Lanka

**Keywords:** hantaviruses, leptospira, sero-prevalence, chronic kidney disease, CKDu

## Abstract

Chronic Kidney disease of uncertain etiology (CKDu) has become a significant disease burden, affecting farming community of Sri Lanka and the exact etiology, which could be multifactorial, is not hitherto established. This study is aimed to determine the association of past hantavirus infection and leptospirosis with the occurrence of CKDu. A cohort (*n* = 179) of known CKDu patients living in high-CKDu prevalent areas of Anuradhapura district of Sri Lanka was compared with a group of 49 healthy, sex-matched younger blood relatives of CKDu patients (control-1) and another 48 healthy, age, and sex-matched individuals living in low-CKDu prevalent area (control-2) of the same district where same life style and climate conditions prevail. Fifty out of 179 (27.9%) CKDu patients, 16/49 (32.7%) of control-1 and 7/48 (14.6%) of control-2 were found positive for IgG antibodies to Puumala, Hantaan or both strains of hantaviruses. Hantaan strain specificity was found to be predominant in all study groups. Hantavirus IgG sero-prevalence of healthy individuals living in low-CKDu prevalent area was significantly lower compared to CKDu patients and healthy younger blood relatives living in high-CKDu prevalent areas (*p* = 0.03). Past hantavirus infection possesses a significant risk for the occurrence of CKDu (OR = 4.5; 95% CI-3.1-5.4, *p* = 0.02). In contrast, IgG seroprevalence to hantaviruses was not significantly different in CKDu patients and healthy younger blood relatives living in high-CKDu prevalent areas indicating past hantavirus infection has no association with the occurrence of CKDu or possibly, younger relatives may develop CKDu in subsequent years. Seroprevalence to leptospirosis showed no significant difference between CKDu patients and healthy controls.

## Introduction

The prevalence of chronic kidney diseases (CKD) is rising in Sri Lanka. Endemic occurrence of kidney disease was first recognized in early 1990s in geographically discrete areas in the dry zone of Sri Lanka, and this has been increasing over past 10–15 years. However, the disease in Sri Lanka is found to be different and not associated with any known risk factors including diabetes, hypertension, or chronic glomerulonephritis (Aturaliya et al., 2006; Agneta et al., 2008; Chandrajith et al., 2010).

Initially, a high prevalence of chronic kidney disease of uncertain etiology (CKDu) is observed in regions of the North Central Province of Sri Lanka (Chandrajith et al., 2010). At present CKDu has spread to other provinces in the dry zone and also into some pockets of the wet zone (Aturaliya et al., 2006). Further, CKDu is commonly seen in paddy farmers and manual laborers who engage in strenuous labor activities in those regions.

Multiple risk factors have been postulated for the development of disease including dehydration, excessive usage of pesticides, past history of leptospirosis, recurrent urinary tract infections, genetic basis, and consumption of mycotoxins (Siriwardhana et al., 2014).^1^ Since most of the victims of CKDu are farmers and CKDu is endemic in the dry zone of Sri Lanka, the relative tendency to development of dehydration is high.

The histological appearance of the disease is “*tubulointerstitial*” that can commonly be observed in toxic nephropathies (Nanayakkara et al., 2011; Weaver et al., 2015; Friesema et al., 2018).^1^ Similar endemic occurrence of CKDu is observed in sugarcane farming community in Nicaragua and South America. CKDu has also been reported in the state of Andhra Pradesh in India and the El-Minia Governorate in Egypt and multiple risk factors have been considered, but the exact etiology is yet to be explored (Nanayakkara et al., 2011; Weaver et al., 2015).

*Orthohantaviruses* are rodent and insectivore-borne viruses of the family *Buniyaviridae* causing two human syndromes namely hemorrhagic fever with renal syndrome (HFRS) in Eurasia and hantavirus cardiopulmonary syndrome (HCPS) in the Americas. Hantaan virus (HTNV) in Asia and Dobrava—Belgrade hantaviruses (DOBVs) in Balkans cause a severe form of HFRS, whereas Puumala virus (PUUV) cause a milder form of HFRS in Europe. Seoul virus (SEOV), carried by urban rats, causes a moderate HFRS worldwide. Sin Nombre (SNV) and Andes (ANDV) viruses are the main causative agents of HCPS in Americas (Jonsson et al., 2010; Vaheri et al., 2011, Vaheri et al., 2013; Klempa et al., 2013). The infection in rodents is chronic and asymptomatic, and infected animals shed the virus in their excreta (Jonsson et al., 2010; Vaheri et al., 2011, Vaheri et al., 2013; Klempa et al., 2013).

In addition to *Orthohantaviruses*, pathogenic spirochetes of the genus *Leptospira* are re-emerging global zoonoses clinically indistinguishable from hantavirus disease in human patients (Friesema et al., 2018).

The disease spectrum of leptospirosis varies from asymptomatic to more severe acute renal failure, pulmonary hemorrhages, sepsis, and death. Rat is the major reservoir of pathogenic Leptospira. Transmission to humans can occur following direct contact with blood, tissues, organs, or urine of infected animals, or through indirect contact, when injured mucosa or skin is exposed to contaminated water (Biruck et al., 2011).

A recent study carried out in the Western Province of Sri Lanka among acutely hospitalized patients with leptospirosis-like illness with kidney involvement has shown that both leptospirosis and hantavirus infections can occur separately as well as co-infections (Sunil-Chandra et al., 2015). In addition, it was reported both PUUV and HTNV specificities in the sera of acutely hospitalized patients and previously hospitalized individuals with past exposure (Sunil-Chandra et al., 2015). Previous exposure to leptospirosis and hantavirus disease could lead to acute renal failure (Sion et al., 2002; Sunil-Chandra et al., 2015). Therefore, the exposure of these pathogens leading to acute kidney injury could act as the initiator of the endemic occurrence of CKDu in Sri Lanka (U.S. Renal Data System: USRDS, 1997; Sion et al., 2002; Jayathilake et al., 2013; Sunil-Chandra et al., 2015). Although it has been hypothesized by various research groups that the hantavirus infection and leptospirosis as possible causes for CKDu in Sri Lanka, to date, the association of these pathogens for the occurrence of CKDu is not clearly elucidated. This study is thus aimed to determine the association of past exposure to hantaviruses or Leptospira or both pathogens and the occurrence of CKDu among CKDu patients and healthy individuals under similar living conditions.

## Materials and Methods

**Figure 1:**
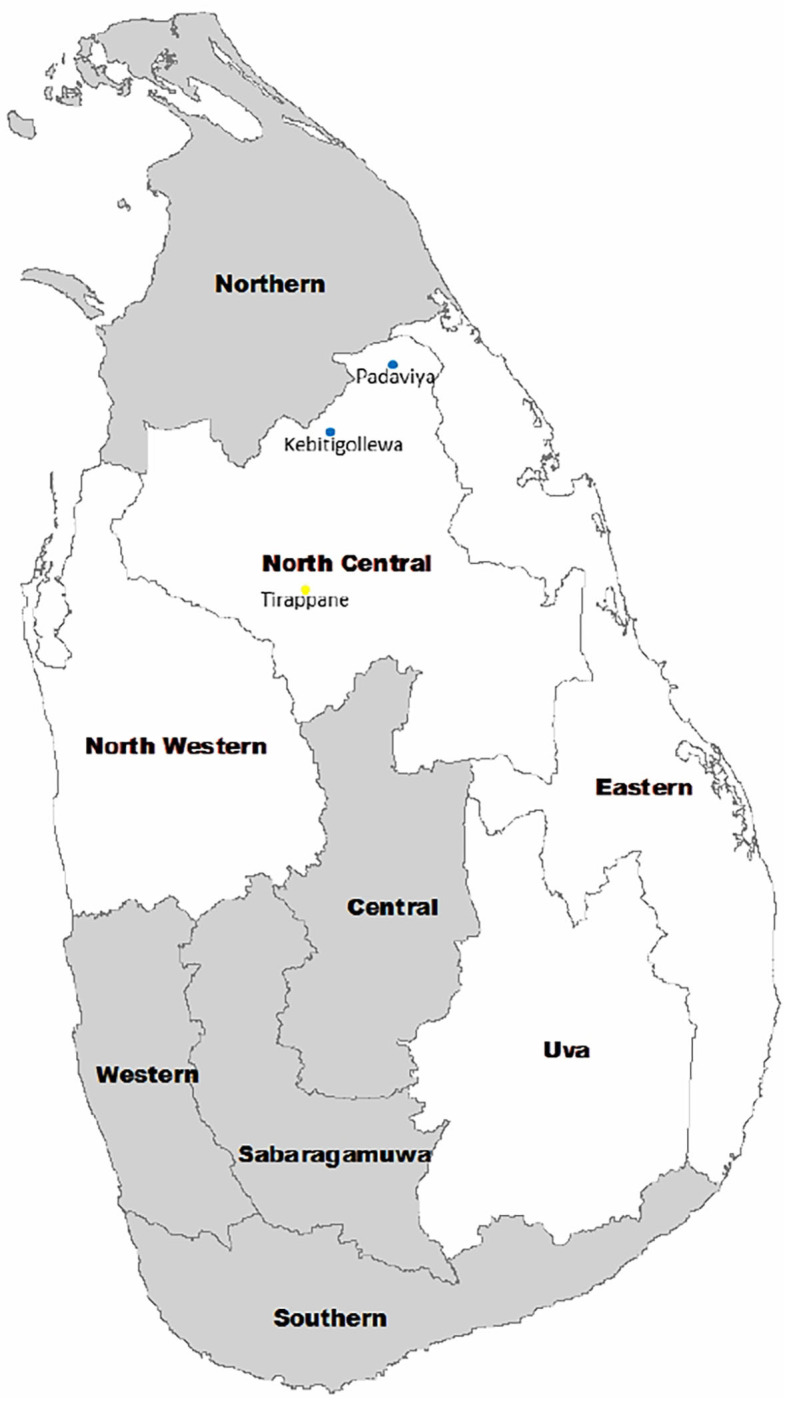
Location of CKDu endemic areas in Sri Lanka. Endemic areas include North Western, North Central, Eastern, and Uva Provinces. CKDu high prevalent areas include Kebitigollewa and Padaviya. Tirappane is the CKDu-low prevalent area.

This was a case-control study carried out with a group of known CKDu patients (cases) living in a high-CKDu prevalent area of Anuradhapura district of the North Central Province of Sri Lanka (Figure 1) and their blood samples were tested for the presence of IgG seroconversion to hantaviruses and Leptospira species. It has been reported previously that the Hantaan and Puumala antigen specificities were detected in sera of hospitalized patients with leptospirosis-like illness in the Western Province of Sri Lanka (Sunil-Chandra et al., 2015). Therefore, in the present study, IgG sero-prevalence to *Hantaan* and *Puumala* strains of hantaviruses and Leptospira in CKDu patients were compared with that of healthy controls 1 (C1) and 2 (C2).

P^H^, physical properties (specific gravity, color), Chemical analysis (Ca^++^, ${\text{PO}}_{4}^{3+}$, Blood Urea) were analyzed in all CKDu samples, including 2 control groups (C1 and C2) in order to determine whether any significant difference is existing. Using the information obtained from patient interviews, questionnaire and clinic documents, the etiological association for CKD was assessed, and those who do not have an exact etiology were considered as CKDu patients.

According to CKDu case definition, an individual identified with an albumin-to-creatinine ratio (ACR) ≥30 mg/g urine creatinine during the initial visit and at a follow-up visit, a normal glycosylated hemoglobin (HbA_1c_ < 6.5%), not on treatment for diabetes, no elevated blood pressure, and no past history of kidney disease or snake bite were included. Patients with other known causes of chronic kidney disease (CKD) and those who did not consent were excluded from the study (U.S. Renal Data System: USRDS, 1997; Biruck et al., 2011). Studies involving human patients/participants were reviewed and approved by the Research, Ethical Review and Higher degree committee of the faculty of Medicine and Allied Sciences, Rajarata University, Sri Lanka. Patients/participants provided their written informed consent to participate in this study (ERC/2012/33).

### Study Subjects

#### CKDu Patients (Cases)

One hundred and seventy-nine (179) CKDu patients on clinic follow up at identified clinical centers of District Hospital, Medawachchiya in the Anuradhapura district were included as cases. All CKDu patients were located in Kebithigollewa and Padaviya divisional secretariat areas where CKDu is highly prevalent in the Anuradhapura district of North Central Province, Sri Lanka and receiving treatment from Renal Treatment Unit, Medawachchiya, Sri Lanka (Figure 1).

#### Healthy Control-1 (C1)

Forty-nine (49) healthy sex-matched individuals having albumin-to-creatinine ratio <30 mg/g in urine were included in the control-1. They were younger blood relatives of CKDu patients, and their living conditions, habits, and locations are almost similar to CKDu patients. All patients were from the Kebithigollewa and Padaviya divisional secretariat areas where CKDu is highly prevalent in the Anuradhapura district of North Central Province, Sri Lanka (Figure 1).

#### Healthy Control-2 (C2)

Forty-eight (48) healthy, age, and sex-matched individuals having albumin creatinine ratio <30 mg/ g in urine were included in the control-2. They were from a CKDu-low prevalent locale in Tirappane divisional secretariat of the Anuradhapura district of the North Central Province, Sri Lanka (Figure 1). Their living conditions and habits were almost similar to Control-1 and test subjects.

### Sample Collection

Since exact etiology for CKDu is not known, a descriptive cross-sectional study with 384 patients had to be included (Prevalence 50%, as prevalence is unknown thus maximum subjects to be included). Due to limitations in funds for detecting seroconversion to hantaviruses and leptospirosis, the present study included 179 CKDu patients, 49 Control-1 individuals, and 48 Control-2 individuals.

A single sample of blood from each individual was collected and later serum was analyzed under sterile and aseptic conditions adhering to standard precautions from a total of 179 CKDu patients, 49 and 48 individuals from control-1 and−2, respectively and serum was separated from each blood sample and stored at −80°C for serology. Serum samples from the CKDu patients (cases), control-1, and−2 were tested for the presence of IgG antibodies specific for Hantavirus-*Hantaan* strain (HTNV), Hantavirus-*Puumala* strain (PUUV) and Leptospira antigens of Genus specificity.

### Tests for IgG Antibodies Specific for Hantaviruses and Leptospira

Sera were tested for the presence of anti-PUUV, anti-HTNV, and anti Leptospira IgG antibodies using commercially available indirect Enzyme-linked immunosorbent assay (Hantaan IgG ELISA, Puumala IgG ELISA, and Leptospira IgG ELISA, Creative diagnostics, USA).

#### ELISA Procedure for Detection of Anti-hantavirus IgG

Indirect Enzyme-linked immunosorbent assay (ELISA) was performed using a recombinant nucleocapsid protein of Puumala strain of Hantavirus (PUUV) for PUUV IgG ELISA and a recombinant nucleoprotein of Hantaan /Dobrava strain of Hantavirus (HTNV) for Hantaan IgG ELISA as the coating antigens to capture IgG antibodies to PUUV and HTNV, respectively. ELISA was performed according to manufacturers' instructions using appropriate positive and negative controls (Creative diagnostics, USA).

Briefly, 96 well microtiter plates pre-coated with recombinant Puumala antigen (N120) or Hantaan antigen (provided in the kit) were allowed to reach the room temperature (25°C). Pipetted 100 μl of undiluted negative, positive, and reference controls as well as 1/20 diluted patients and control individuals' sera per well. Covered wells with adhesive foil and incubated at 37°C for 45 min. Emptied wells and filled each well with 300 μl wash buffer and emptied wells again. Repeated this wash three more times. Removed excess liquid by tapping the strips onto absorbent paper. Pipetted 100 μl of ready to use anti-IgG peroxidase conjugate (1x CG) per well, covered wells with adhesive foil and incubated at 37°C for 45 min. Washed wells four times with 300 μl wash buffer per well again, added 100 μl 3,3′,5,5′-tetramethylbenzidine (TMB) substrate solution per well and incubated 10 min at room temperature (25°C). After the addition of 100 μl per well of 0.25M H_2_SO_4_ stop solution, measured color (optical density) within 20 min at 450 nm (reference wavelength at 650 nm using an ELISA reader (Biotek-Ex800).

#### ELISA Procedure for Detection of Anti-leptospira IgG

A commercially available enzyme Immunoassay for the determination of IgG antibodies to Genus specific *Leptospira* antigens (Creative Diagnostics, USA) was performed and results were interpreted according to manufacturer's instructions.

Briefly, 96 well microtiter plates pre-coated with Genus specific Leptospira antigens were allowed to reach the room temperature (25°C). Pipetted 100 μl of undiluted ready to use negative, positive, and reference control sera as well as 100 μl/well of 1/100 diluted patients' or control individuals' (C1 and C2) sera. Spared one well for substrate blank. Covered wells with adhesive foil and incubated at 37°C for 60 min in a moist chamber. After incubation, emptied wells and filled each well with 300 μl wash buffer and emptied wells again.

Repeated this wash three more times (altogether 4 times). Removed excess liquid by tapping the strips onto absorbent paper. Pipetted 100 μl of ready to use anti-IgG peroxidase conjugate per well, covered wells with adhesive foil and incubated at 37°C for 30 min in moist chamber. After incubation, washed all wells four times with 300 μl wash buffer per well again, added 100 μl 3,3′,5,5′-tetramethylbenzidine (TMB) substrate solution per well including well for substrate blank and incubated 30 min at room 37°C in moist chamber.

After the addition of 100 μl per well of 0.25M H_2_SO_4_ stop solution, shake gently to mix and measured optical density within 60 min at 405 nm (reference wavelength at 650 nm using an ELISA reader (Biotek-Ex800).

#### Evaluation and Calculation of ELISA Absorbance

Absorbance of test samples by HTNV IgG ELISA and PUUV IgG ELISA was measured as optical density (OD) and the results were calculated by obtaining the Q value which is the ratio of the OD values of the test sample over Reference control sample. If the Q is <1 the sample is considered as negative for IgG antibodies. If it is 1 ≤ Q ≤ 1.5 the sample is considered as equivocal. If the Q is > 1.5 the sample is considered as positive.

Absorbance of test samples by the Leptospira IgG ELISA was measured as optical density (OD) and a sample is considered positive if the sample is having >11 ELISA units which is the ratio of the OD values of the test sample over Reference control sample × 10. Sample is considered as equivocal if it gives 9–11 ELISA units. Sample is considered as negative if it gives <9 ELISA units.

### Statistical Analysis

Continuous data were expressed in measures of central tendency. Association between categorical variables was assessed using the chi-squared test, and risk factor analysis was performed using conditional logistic regression (1:2 age and sex-matched). Variables showing statistically significant association in univariate analysis with the outcome variable were considered as a risk factor. Only those variables were subjected to multivariate analysis. SAS Statistical software was used for the analysis (SAS Institute, 2005).

## Results

### Demography and Social Status of Cases and Controls

One hundred and seventy-nine (179) CKDu cases, 49 control-1 and 48 control-2 were included in the study. The age distribution of CKDu cases was 44.1 ± 9.9 years (mean ± standard deviation) while control-1 and control-2 were 31.8 ± 4.7 years and 44.2 ± 10.8 years, respectively. The duration of CKDu was 8.5 ± 4.5 years. The gender distribution of CKDu cases, control-1 and control-2, was (male:female) 2:1, 2:1, and 2:1, respectively (Table 1). Majority were farmers (CKDu cases−69%, control 1−53%, and control 2−69%) whilst monthly income per person in CKDu cases, control-1 and control-2 was LKR 9,000 ± 2,200, 9,200 ± 2,100, and 8,900 ± 2,200, respectively. Almost all of CKDu, C1 and C2 groups have been exposed to rodents irrespective of the age or locality amounting to 173/179 (97%), 47/49 (96%), and 46/48 (96%), respectively.

**Table 1:** Study population and setting.

**Study group**	**Age range in years (mean)**	**Number studied (status)**	**PROVINCE District *Division/s***
CKDu patients (ACR ≥ 30 mg/g urine)	29–67 (44.1 ± 9.9)	179 (clinic attendees live in high prevalent areas)	NORTH CENTRAL Anuradhapura 1. *Kebithigollewa* 2. *Padaviya*
Healthy control group 1 (ACR < 30 mg/g urine)	21–44 (31.8 ± 4.7)	49 (healthy blood relatives live in high prevalent areas)	NORTH CENTRAL Anuradhapura 1. *Kebithigollewa* 2. *Padaviya*
Healthy control group 2 (ACR < 30 mg/g urine)	29–67 (44.2 ± 10.8)	48 (healthy non-relatives live in low prevalent area)	NORTH CENTRAL Anuradhapura 1. *Tirappane*

### Hantavirus Antibody Positivity

**Figure 2:**
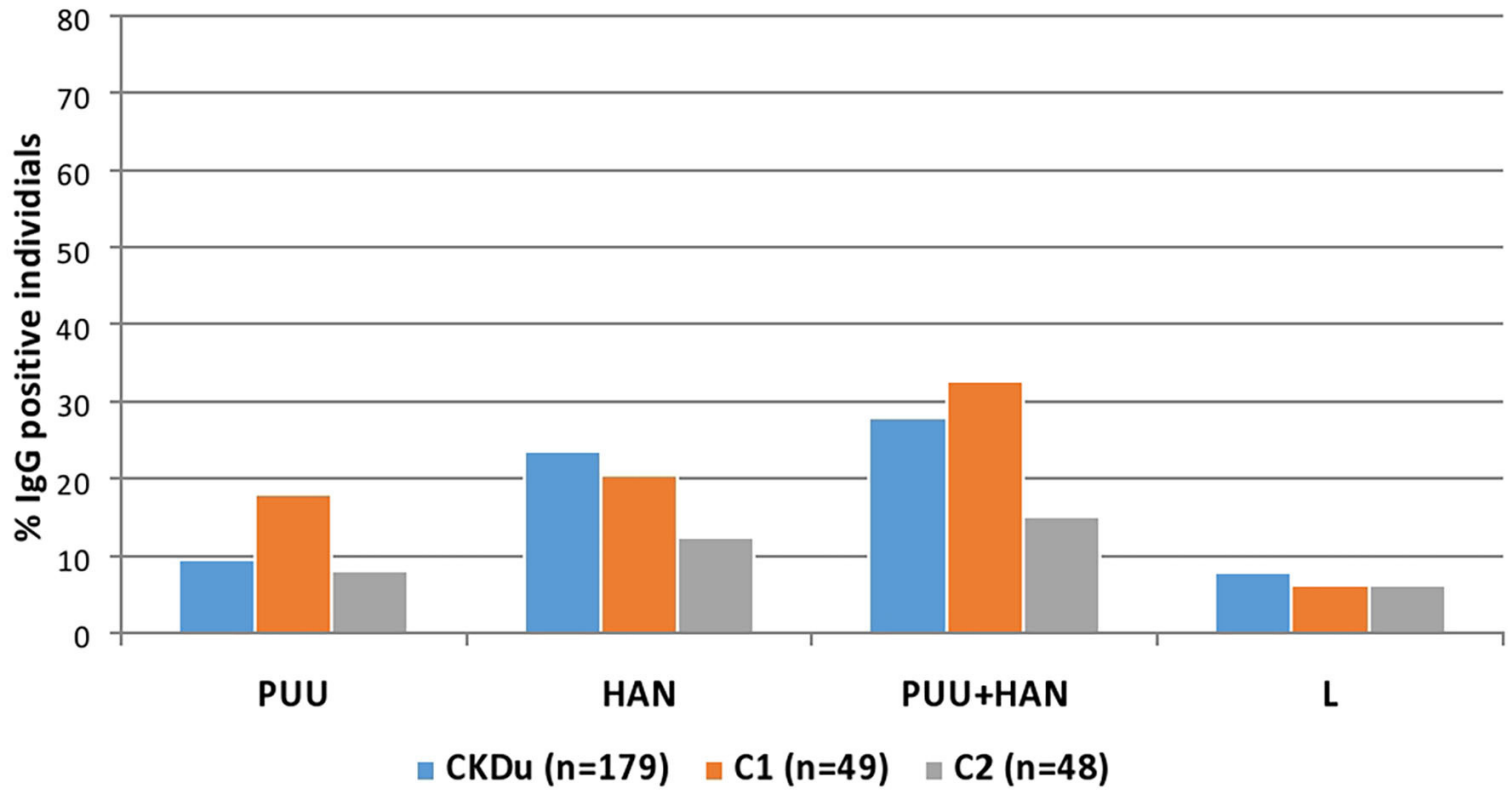
Hantavirus and Leptospira specific IgG in CKDu patients and healthy control groups; C1 and C2. CKDu: Chronic Kidney disease patients in high-CKDu prevalent area, C1: healthy blood relatives in high-CKDu prevalent area, C2: healthy individuals from low-CKDu prevalent area. PUU: Puumala strain specificity, HAN: Hantaan strain specificity, PUU+HAN: Total number positive for either Hantaan, Puumala and both strains, L: Leptospira specificity.

**Figure 3:**
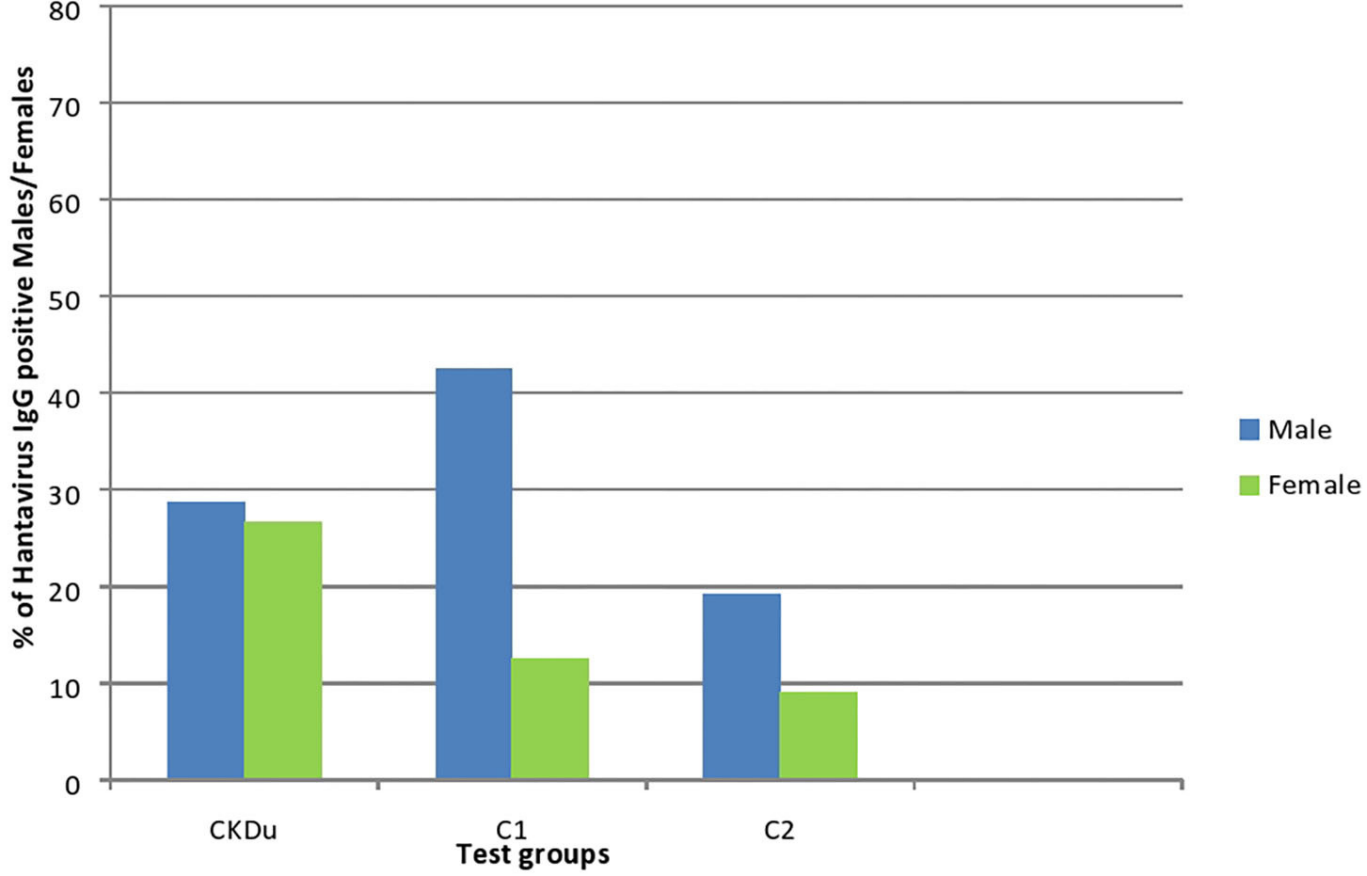
Gender distribution and exposure to hantaviruses among CKDu patients and healthy controls; C1 and C2. CKDu: Chronic Kidney disease patients in high-CKDu prevalent area, C1: healthy blood relatives in high-CKDu prevalent area, C2: healthy individuals from low-CKDu prevalent area.

Of 50/179 (27.9%) CKDu cases, 16/49 (32.7%) of the control-1 and 7/48 (14.6%) control-2 were positive for IgG antibodies to *Puumala* or *Hantaan* strains or both strains (Table 2, Figures 2, 3). Compared to control-2, Collective positivity for *Puumala*, and *Hantaan* IgG antibodies is significantly high in CKDu patients (chi-square = 4.851 *p* = 0.02763). Further, the results showed that the Hantaan strain specificity was found to be predominant in all study groups (Table 2, Figure 2). Although the exposures to both Hantaan and Puumala strains of hantaviruses have been observed in CKDu patients and healthy controls in the present study, individuals positive for Hantaan strain specificity was found to be the predominant in all study groups (23.5% in CKDu, 20.4% in C1 and 12.5% in C2) indicating that the Hantaan strain harboring rodent species are the principal animal reservoir in the study areas (Table 2).

**Table 2:** Past exposure to hantaviruses and leptospirosis among CKDu patients and healthy controls.

**IgG antibody specificity**	**CKDu patients (*n* = 179)**	**Control 1 (*n* = 49)**	**Control 2 (*n* = 48)**
PUU strain specific IgG positive individuals	17/179 (9.5%)	9/49 (18.4%)	4/48 (8.3%)
Hantaan strain specific IgG positive individuals	42/179 (23.5%)	10/49 (20.4%)	6/48 (12.5%)
Total Hantavirus Specific IgG positive individuals (PUU specific IgG + Hantaan specific IgG+ PUU and Hantaan cross reactive IgG)	50/179 (27.9%)	16/49 (32.7%)	7/48 (14.6%)
Leptospira specific IgG positive individuals	14/179 (7.8%)	3/49 (6.1%)	3/48 (6.3%)

### Leptospira Antibody Positivity

**Figure 4:**
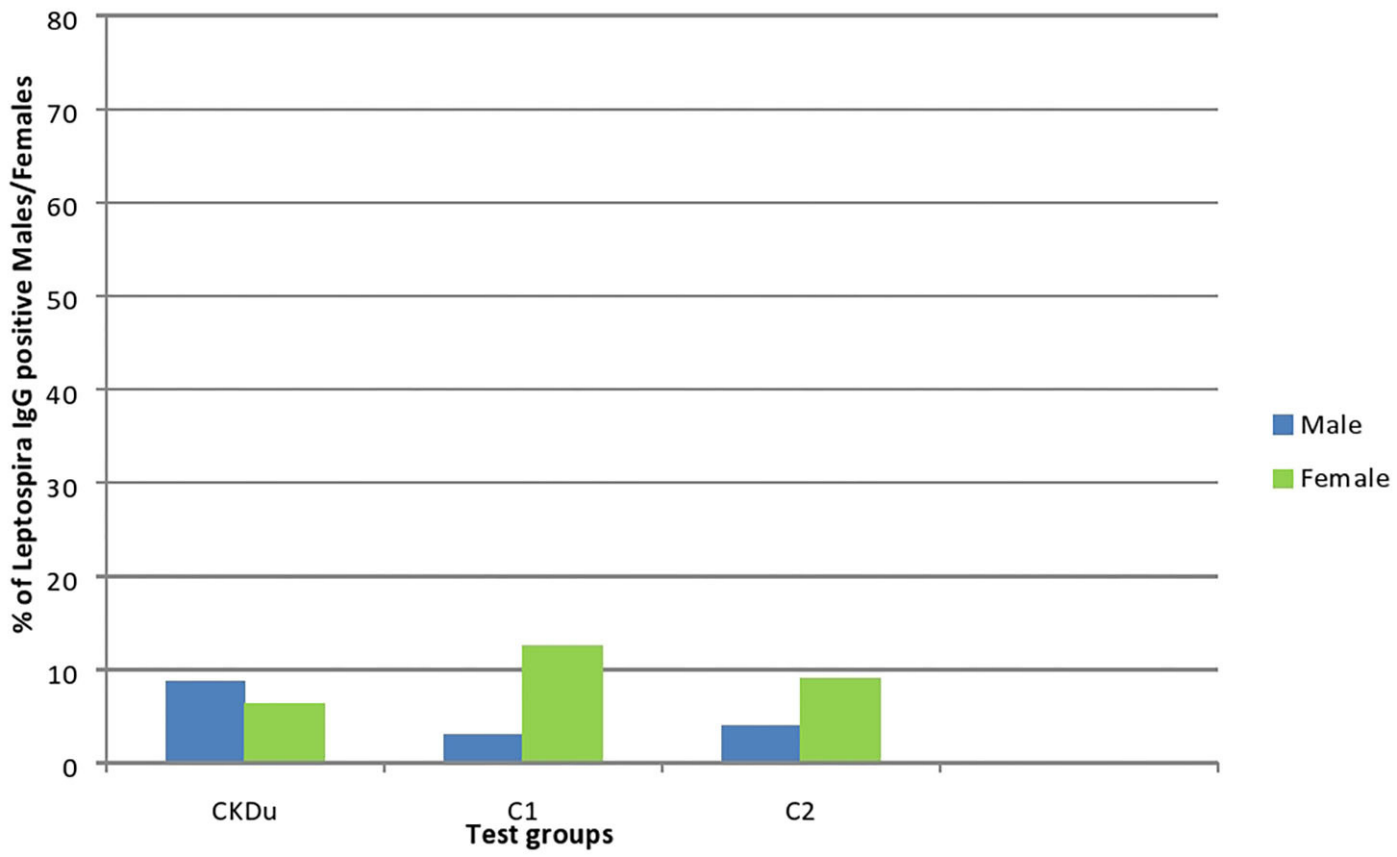
Gender distribution and exposure to Leptospira among CKDu patients and healthy Controls; C1 and C2. CKDu: Chronic Kidney disease patients in high-CKDu prevalent area, C1: healthy blood relatives in high-CKDu prevalent area, C2: healthy individuals from low-CKDu prevalent area. Overall exposure to leptospirosis was similar in CKDu patients (7.8%), C1 (6.1%), and C2 (6.3%) but higher exposure was observed in females (12.5% in C1 and 9.1% in C2) compared to males (3% in C1 and 3.9% in C2) in healthy controls.

**Figure 5:**
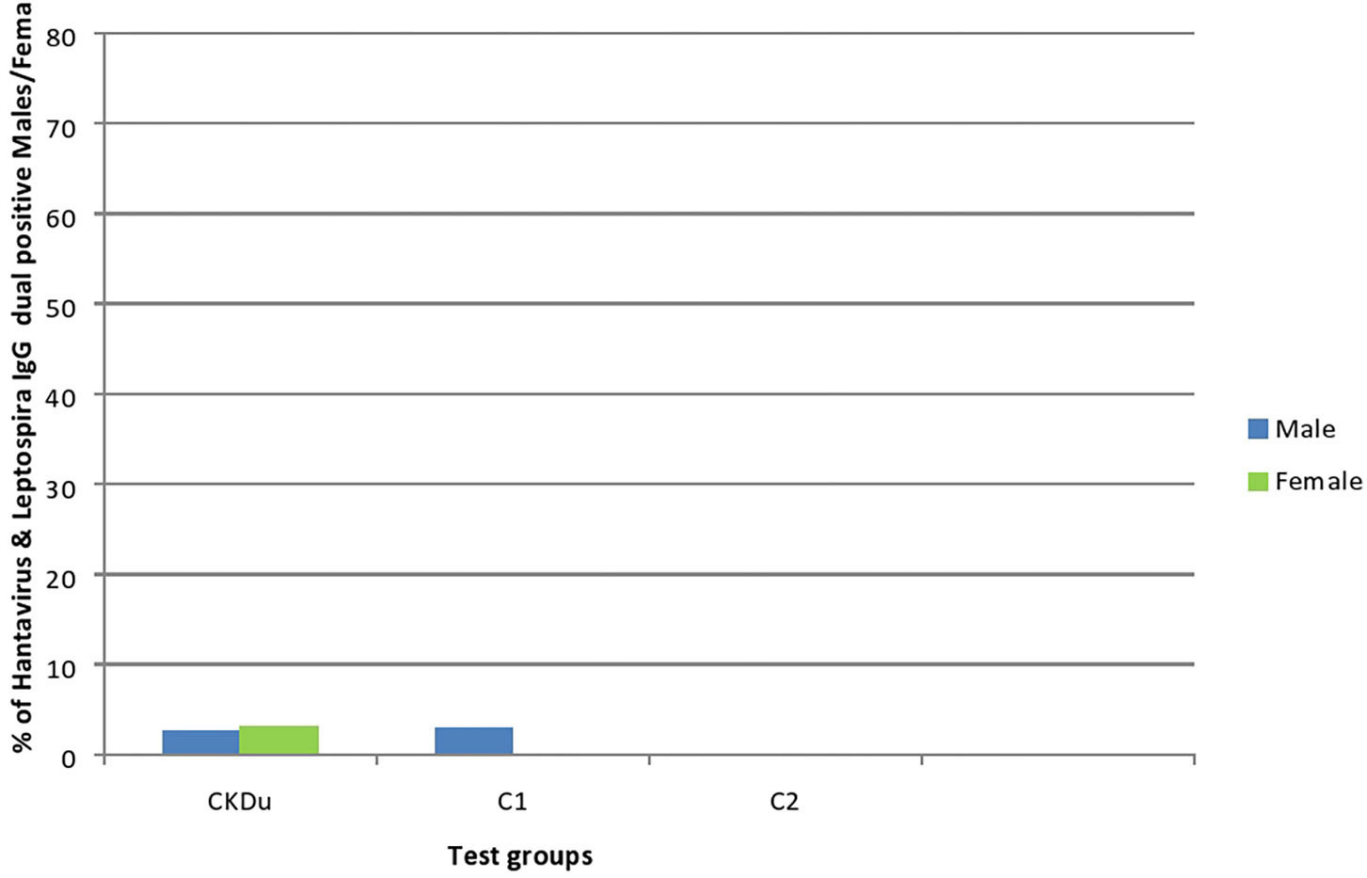
Gender distribution and dual exposure to Hantaviruses and Leptospira among CKDu patients and healthy controls; C1 and C2. CKDu: Chronic Kidney disease patients in high-CKDu prevalent area, C1: healthy blood relatives in high-CKDu prevalent area, C2: healthy individuals from low-CKDu prevalent area. Low levels of dual exposures to hantaviruses and leptospira or co-infections have occurred in high-CKdu prevalent areas (2.8% in CKDu patients 2% in C1-healthy blood relatives) compared to healthy control -C2 (0%) in low-CKDu prevalent area.

Only 14/179 (7.8%) CKDu patients, 3/49 (6.1%) control-1 and 3/48 (6.3%) control-2 had IgG antibodies to Leptospira, respectively (Table 2, Figures 4, 5). The leptospirosis IgG antibody positivity was not significantly (*p* > 0.05) differed among three groups.

### The Risk Factors for the Development of CKDu

Rodents are worldwide reservoirs for both hantaviruses and Leptospira and in Sri Lanka too. In the present study, majority of CKDu patients and healthy control 1 and 2 groups are farmers and almost all have been exposed to rodents irrespective of age or locality with an occupational risk of exposing to hantaviruses and Leptospira to rodents or their excreta.

Among the postulated multiple risk factors, past exposure to hantavirus infections is a significant risk factor for the development of CKDu (*OR* = 4.5; 95% CI-3.1-5.4, *p* = 0.02). None of them has developed acute leptospirosis-like illness warranting hospital admission during the study period.

#### Seroconversion to Hantaviruses With Age and Gender Distribution

**Figure 6:**
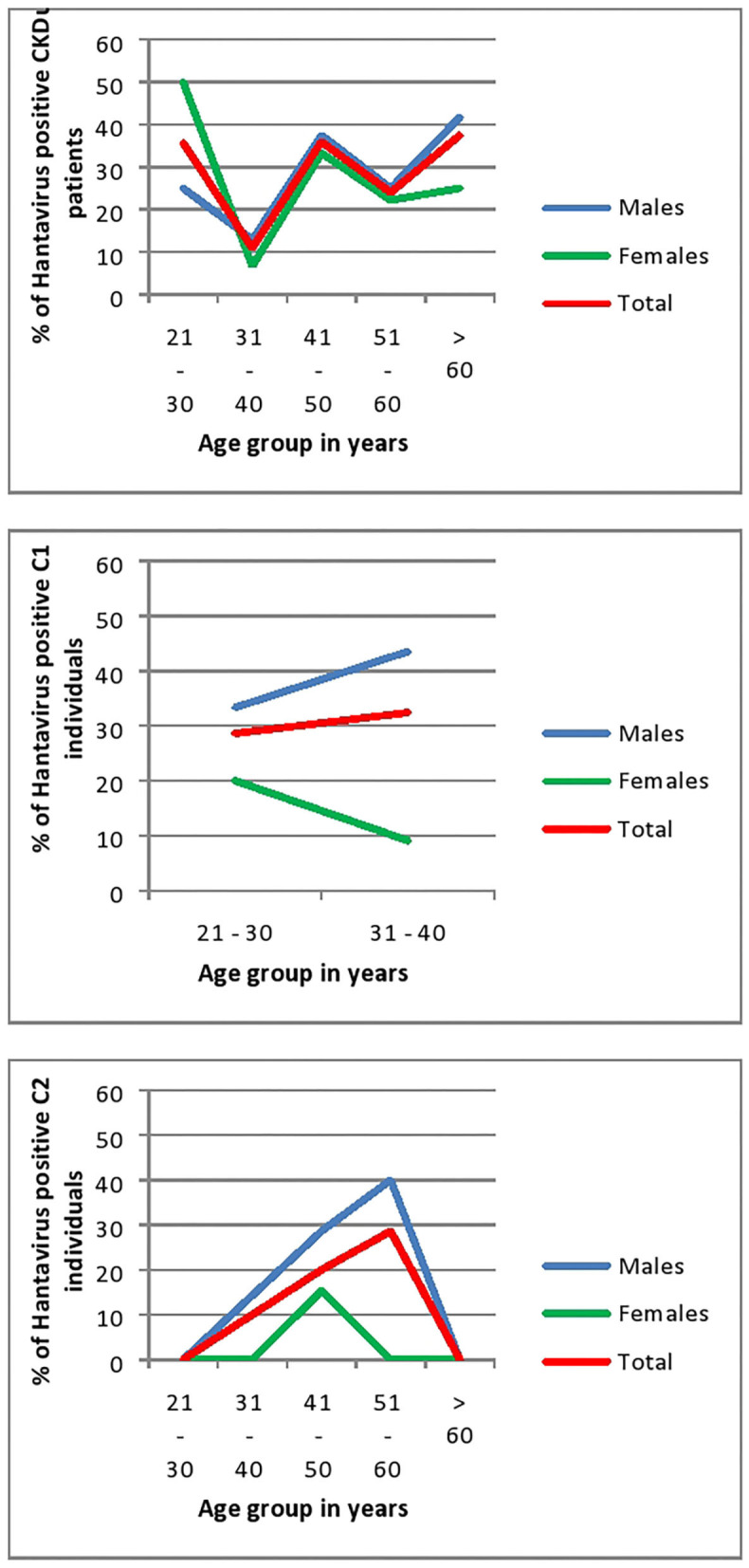
Age and Gender related seroconversion to hantaviruses in CKDu patients and healthy controls; C1 (younger relatives) and C2. CKDu: Chronic Kidney disease patients in high-CKDu prevalent area, C1: healthy younger relatives in high-CKDu prevalent area, C2: healthy individuals from low-CKDu prevalent area.

In all age groups of CKDu patients except the 21–30 age group males had higher seroconversion to hantaviruses than females. Rising of seroconversion with age is observed in male patients with CKDu. Control 1 and Control 2 also have shown a rising of seroconversion with age (Figure 6). Males had higher seroconversion compared to females in control groups.

As a whole, there is higher but similar exposures to hantaviruses observed in both male (28.7%) and female (26.6%) CKDu patients but only males (42.4% in C1 and 19.2% in C2) have higher exposure compared to females (12.5% in C1 and 9.1% in C2) in healthy controls (Figure 3).

In CKDu and control-1 groups, anti-hantavirus IgG antibody positivity is significantly high in both 21–30 years (*p* = 0.03) and 31–40 years' age categories (*p* = 0.03) and showed a rising trend with the age. Control-2 group also showed a rising trend of anti-hantavirus IgG antibody positivity with age (Figure 6).

## Discussion

The possible etiology of CKDu is predominantly predicted with connection of the usage of agrochemicals (Jayasumana et al., 2015). However, the assessment of past infections by hantaviruses, leptospira, asymptomatic bacteriuria in CKDu patients would be important to establish the association of infective etiology for CKDu. Another group of researchers has previously investigated the possibility of hantavirus infection as a risk factor for the occurrence of CKDu in Sri Lanka (Gamage et al., 2017; Sarathkumara et al., 2019).

In contrast to the previous work (Gamage et al., 2017; Sarathkumara et al., 2019), here we have studied the past exposure to both hantavirus infections and leptospirosis in CKDu patients from high CKDu prevalent areas with that of healthy persons living in both high and low CKDu prevalent areas in the same district; notably with same living and environment conditions.

Since most of people were having CKDu in the CKDu high prevalent area, finding an age matched healthy control from CKDu high prevalent area was very difficult.

Although there was an increasing trend for seroconversion to hantaviruses in male CKDu patients, the overall seroprevalence to hantavirus was similar in both males and females (Figure 6).

Hantavirus IgG seroprevalence of healthy individuals living in CKDu low prevalent area (control 2) was significantly low compared to the seroprevalence of CKDu patients and their healthy younger blood relatives (control-1) living in CKDu high prevalent areas (*p* = 0.03) who share same living and environmental conditions (Table 2, Figures 2, 3).

Alarmingly high hantavirus IgG seroprevalences of 28.6 and 32.4% were detected among healthy young blood relatives of age groups 21–30 and 31–40 years living in CKDu high prevalent areas, respectively. It would be an early indication of developing CKDu in the future among current young and healthy population.

As this study was aimed to determine the relationship of seroconversion to hantaviruses and Leptospira with the occurrence of CKD of unknown etiology (CKDu), patients with CKD due to known causes such as diabetes and hypertension were excluded. As a result, age related diseases such as diabetes, hypertension could not be studied in CKDu patients. Therefore, future studies should be focused on other age-related diseases among CKDu patients in order to explain the impact of age on other diseases in the study areas.

There could have a genetic component for the occurrence of CKDu that could arise over certain age but with the limited research funding of the present study only the demographic data and seroconversion to hantaviruses and Leptospira were included.

In a parallel study, the urine cultures obtained at different time intervals from CKDu patients and healthy controls were found to be negative for significant bacteriuria with colony count of <10^3^ CFU/ml indicated that asymptomatic bacteriuria is not a potential risk factor for the development of CKDu in the farming community (Jayaweera, unpublished observations).

Present study indicates that the past hantavirus infections are a significant risk factor for the occurrence of CKDu in the North Central Province of Sri Lanka. Since all most all the individuals of CKDu, C1, and C2 groups have been exposed to rodents irrespective of age or locality amounting to 173/179 (97%), 47/49 (96%), and 46/48 (96%), respectively the relative abundance of hantavirus carrying rodents in those areas may determine the frequency of exposure to hantaviruses and leptospirosis. Therefore, it can be speculated that the rodents of CKDu high prevalent areas may have been carrying hantaviruses than rodents in CKDu low prevalent areas. Future studies should be carried out to determine the seroprevalence of hantaviruses in the rodent populations of CKDu high and low prevalent areas and also a follow up study on hantavirus exposed healthy younger relatives for the development of CKDu.

According to risk factor analysis, only the past hantavirus infection possesses a significant risk for the development of CKDu in the people who live in CKDu highly prevalent areas compared to those live in low prevalent area. However, we assume that the etiology of CKDu could be collectively multifactorial in origin including hantavirus infections amongst the farming community.

Hantavirus infection is a well-known cause for acute kidney injury (Jonsson et al., 2010). Therefore, our proposed mechanism for pathophysiology of CKDu in Sri Lanka is that past hantavirus infections in renal parenchyma would act as the nidus and subsequently when people become exposed to other risk factors (i.e., pesticide exposure and dehydration) it could lead to the development of CKDu.

In conclusion, past hantavirus infection was significantly associated with the occurrence of CKDu (*OR* = 4.5; 95% CI-3.1-5.4, *p* = 0.02) indicating a possible future risk of developing of CKDu in healthy young individuals living in CKDu high prevalent areas. Further, the past leptospirosis is not significantly associated (*p* > 0.05) with the occurrence of CKDu. The present study also reveals that there is a rising trend of anti-hantavirus IgG seroconversion with age.

## Data Availability Statement

All datasets presented in this study are included in the article/supplementary material.

## Ethics Statement

The studies involving human participants were reviewed and approved by Research, Ethical Review, and Higher Degrees Committee of the Faculty of Medicine and Allied Sciences, Rajarata University, Saliyapura, Sri Lanka. The patients/participants provided their written informed consent to participate in this study.

## Author Contributions

NS-C and JJ conceived the study and NS-C received funding for the study. JJ and WK carried out the sampling and collection of patient data. NS-C, JJ, and MJ analyzed the samples. NS-C wrote the draft of the manuscript. All authors participated in discussions affecting the contents and direction of the manuscript. All authors have read and approved the final manuscript.

## Conflict of Interest

The authors declare that the research was conducted in the absence of any commercial or financial relationships that could be construed as a potential conflict of interest.

## Footnotes

^1^https://laislafoundation.org/american-journal-kidney-disease-publishes-review-ckdu-research-2/ (accessed May 21, 2016).

## References

[B1] Agneta A. P. Wang M. Wengstrom Y. (2008). The impact of a nurse-led clinic on self-care ability, disease-specific knowledge, and home dialysis modality. Nephrol. Nurs. J. 35, 102–106. 18649584

[B2] Aturaliya T. N. C. Abeysekera D. T. D. J. Amerasinghe P. H. Kumarasiri P. V. Bandara P. (2006). “Towards understanding of chronic kidney disease of North Central Province,” in Proceedings of Annual Scientific Sessions of Sri Lanka Medical Association (Colombo).

[B3] Biruck D. Nanayakkara S. Harada K. Hitomi T. Chandrajith R. Karunarathne U.. (2011). Myotoxin detection in urine samples from patients with chronic kidney disease of uncertain etiology in Sri Lanka. Bull. Environ. Contam. Toxicol. 87, 6–10. 10.1007/s00128-011-0301-4 21553028

[B4] Chandrajith R. Nanayakkara S. Itai K. Aturaliya T. N. C. Dissanayake C. B. Abeysekera T.. (2010). Chronic kidney diseases of uncertain etiology (CKDue) in Sri Lanka: geographic distribution and environmental implications. Environ. Geochem. Health 33, 267–278. 10.1007/s10653-010-9339-1 20853020

[B5] Friesema I. H. M. Bakker J. Maas M. Goris M. G. A. van der Giessen J. W. B. Rockx B. H. G. (2018). Seroprevalence of hantaviruses and leptospira in muskrat and coypu trappers in the Netherlands, 2016. Infect. Ecology Epidemiol. 8:1474707. 10.1080/20008686.2018.1474707 29805784 PMC5965032

[B6] Gamage C. D. Yoshimatsu K. Sarathkumara Y. D. Kulendiran T. Nanayakkara N. Arikawa J. (2017). Serological evidence of hantavirus infection in Girandurukotte, an area endemic for chronic kidney disease of unknown aetiology (CKDu) in Sri Lanka. Int. J. Infect. Dis. 57, 77–78. 10.1016/j.ijid.2017.02.004 28212862

[B7] Jayasumana C. Paranagama P. Agampodi S. Wijewardane C. Gunatilake S. Siribaddana S. (2015). Drinking well water and occupational exposure to herbicides is associated with chronic kidney disease, in Padavi-Sripura, Sri Lanka. Environ. Health 14:6. 10.1186/1476-069X-14-6 25596925 PMC4417209

[B8] Jayathilake N. Mendis S. Maheepala P. Mehta F. R. (2013). Chronic kidney disease of uncertain aetiology: prevalence and causative factors in a developing country. BMC Nephrol. 14:180. 10.1186/1471-2369-14-180 23981540 PMC3765913

[B9] Jonsson C. B. Figueiredo L. T. Vapalahti O. (2010). A global perspective on hantavirus ecology, epidemiology, and disease. Clin. Microbiol. Rev. 23, 412–441. 10.1128/CMR.00062-09 20375360 PMC2863364

[B10] Klempa B. Radosa L. Kruger D. H. (2013). The broad spectrum of hantaviruses and their hosts in central europe. Acta Virol. 57, 130–137. 10.4149/av_2013_02_130 23600871

[B11] Nanayakkara S. Komiya T. Ratnatunga N. Senevirathna S. T. Harada K. H. Hitomi T.. (2011). Tubulointerstitial damage as the major pathological lesion in endemic chronic kidney disease among farmers in North Central Province of Sri Lanka. Environ. Health Prev. Med. 17, 213–221. 10.1007/s12199-011-0243-9 21993948 PMC3348245

[B12] Sarathkumara Y. D. Gamage C. D. Lokupathirage S. Muthusinghe D. S. Nanayakkara N. Gunarathne L. (2019). Exposure to hantavirus is a risk factor associated with kidney diseases in Sri Lanka: a cross sectional study. Viruses 11:700 10.3390/v11080700 PMC6723923 31370348

[B13] SAS Institute (2005). SAS^®^ 9.1.3 Language Reference: Concepts, 3rd Edn. Cary, NC: SAS Institute Inc.

[B14] Sion M. L. Hatzitolios A. I. Armenaka M. C. Toulis E. N. Kalampalika D. Mikoudi K. D. (2002). Acute renal failure caused by leptospirosis and hantavirus infection in an urban hospital. Eur. J. Intern. Med. 13, 264–268. 10.1016/S0953-6205(02)00037-7 12067823

[B15] Siriwardhana E. A. R. I. E. Perera P. A. J. Sivakanesan R. Abeysekara T. Nugegoda D. B. Jayaweera J. A. A. S. (2014). Dehydration and malaria in augmenting the risk of developing chronic kidney disease in Sri Lanka. Indian J. Nephrol. 25, 146–151. 10.4103/0971-4065.140712 26060363 PMC4446918

[B16] Sunil-Chandra N. P. Clement J. Maes P. De Silva H. J. Van Esbroeck M. Van Ranst M. (2015). Concomitant leptospirosis – hantavirus co-infections in acute patients hospitalized in Sri Lanka: implications for potentially worldwide underestimated problem. Epidemiol. Infect. 143, 2081–2093. 10.1017/S0950268814003707 25582980 PMC9506992

[B17] U.S. Renal Data System: USRDS (1997). 1997 Annual Data Report. Bethesda, MD: National Institutes of Health, National Institute of Diabetes and Digestive and Kidney Diseases.

[B18] Vaheri A. Mills J. N. Spiropoulou C. F. Hjelle B. (2011). “Hantaviruses,” in Oxford Textbook of Zoonoses: Biology, Clinical Practice, and Public Health Control, 2nd Edn, eds Palmer S. R. Soulsby L. Brown D. Torgerson P. (Oxford: Oxford University Press), 307–322. 10.1093/med/9780198570028.003.0035

[B19] Vaheri A. Strandin T. Hepojoki J. Sironen T. Henttonen H. Makela S.. (2013). Uncovering the mysteries of hantavirus infections. Nat. Rev. Microbiol. 11, 539–550. 10.1038/nrmicro3066 24020072

[B20] Weaver V. M. Fadrowski J. J. Jaar B. G. (2015). Global dimensions of chronic kidney disease of unknown etiology (CKDu): a modern era environmental and/or occupational nephropathy? BMC Nephrol. 16:145 10.1186/s12882-015-0105-6 26282933 PMC4539684

