# Corrigendum: Association of Hantavirus Infections and Leptospirosis With the Occurrence of Chronic Kidney Disease of Uncertain Etiology in the North Central Province of Sri Lanka: A Prospective Study With Patients and Healthy Persons

**DOI:** 10.3389/fcimb.2020.631515

**Published:** 2020-12-17

**Authors:** N. P. Sunil-Chandra, J. A. A. S. Jayaweera, W. Kumbukgolla, M. V. M. L. Jayasundara

**Affiliations:** ^1^Department of Medical Microbiology, Faculty of Medicine, University of Kelaniya, Kelaniya, Sri Lanka; ^2^Faculty of Medicine & Allied Sciences, Rajarata University of Sri Lanka, Anuradhapura, Sri Lanka

**Keywords:** hantaviruses, leptospira, sero-prevalence, chronic kidney disease, CKDu

In the original article, there was an error in the ethical clearance number. The correct number for ethical clearance is **ERC/2012/33**.

A correction has been made to **Materials and Methods**, *Paragraph 3:*

*According to CKDu case definition, an individual identified with an albumin-to-creatinine ratio (ACR) ≥ 30 mg/g urine creatinine during the initial visit and at a follow-up visit, a normal glycosylated hemoglobin (HbA_1c_ <6.5%), not on treatment for diabetes, no elevated blood pressure, and no past history of kidney disease or snake bite were included. Patients with other known causes of chronic kidney disease (CKD) and those who did not consent were excluded from the study (16,13). Studies involving human patients/participants were reviewed and approved by the Research, Ethical Review and Higher degree committee of the faculty of Medicine and Allied Sciences, Rajarata University, Sri Lanka. Patients/participants provided their written informed consent to participate in this study (ERC/2012/33)*.

The authors apologize for this error and state that this does not change the scientific conclusions of the article in any way. The original article has been updated.

